# Intestinal Flora: A Potential Mechanism by Which *Yinlai* Decoction Treats Lipopolysaccharide-Induced Pneumonia

**DOI:** 10.1155/2022/3034714

**Published:** 2022-03-23

**Authors:** Jingnan Xu, Xueyan Ma, Chen Bai, Xin Jiang, Ling Huang, Fei Gao, Yini Li, He Yu, Tiegang Liu, Xiaohong Gu

**Affiliations:** Beijing University of Chinese Medicine, Beijing 100029, China

## Abstract

**Background:**

We intended to explore the mechanism of *Yinlai* decoction in the treatment of lipopolysaccharide (LPS)-induced pneumonia from the perspective of intestinal flora.

**Methods:**

Thirty Sprague–Dawley rats were randomly assigned to the blank control group (N), the pneumonia group (P), and the *Yinlai* decoction group (PT). The rat pneumonia model was established using LPS inhalation (0.5 mg/mL, 5 mL, 30 min/day, 3 days). *Yinlai* decoction was administered intragastrically (2 mL/100 g, 3 days). Lung tissue pathology, organ indexes, serum inflammatory factors, tumor necrosis factor-alpha (TNF-*α*), and intestinal flora changes were measured.

**Results:**

Lung tissue inflammation was prevented by *Yinlai* decoction. IL-6 levels showed a higher tendency to be higher, and IL-12 and TNF-*α* were significantly higher in the PT group than in the P group. The structure of the intestinal flora in the P differed from that in the N. The relative abundance of 10 out of 12 microflora was significantly higher in the P group than in the N and PT groups. In the PT group, the structure and the distribution of microbial groups were like those of the N group.

**Conclusions:**

*Yinlai* decoction inhibited LPS-induced lung and systemic inflammation in rats and may help the intestinal flora restore equilibrium by inhibiting the colonization of pathogenic bacteria and adjusting the ratio between probiotics and pathogenic bacteria. Intestinal flora may serve as a mediator of *Yinlai* decoction's effect on LPS-induced pneumonia.

## 1. Background

Pneumonia is one of the most common diseases of the respiratory system. There are 3.5 million deaths due to pneumonia worldwide yearly, of which about 1.3 million occur in children. The incidence of pneumonia in children under 5 years old is about 120 million per year worldwide, and nearly 12 million children are hospitalized for severe pneumonia [1]. Because of the delay in identifying and diagnosing the pathogens, there are difficulties associated with targeted antibacterial therapy. Therefore, the choice of antibiotics, especially penicillins, cephalosporins, and macrolide antibiotics, is based on clinical experience. Increased bacterial resistance complicates the treatment of pneumonia, and the abuse of antibiotics adversely affects the development of microflora in children [2, 3].

Traditional Chinese medicine (TCM) has been used to treat pneumonia in China for thousands of years and is used in many other countries. The integration of traditional Chinese and Western medicine to treat community-acquired pneumonia significantly shortened hospital stay and reduced mortality [4, 5]. *Yinlai* decoction is composed of *Lonicerae Japonicae Flos* (Jinyinhua), *Raphani Semen* (Laifuzi), *Forsythiae Fructus* (Lianqiao), *Scutellariae Radix* (Huangqin), *Peucedani Radix* (Qianhu), *Houttuyniae Herba* (Yuxingcao), and *Trichosanthes Kirilowii Maxim* (Gualou). It has been used successfully to treat pneumonia in children. *Yinlai* decoction also helped treat the common cold in children [6], and altered the expression of proinflammatory and anti-inflammatory factors in mice's food accumulation and FM1 influenza virus [7]. Relevant targets and immune inflammation pathways were regulated by these herbs to achieve an anti-inflammatory effect and ultimately improve pulmonary inflammation [8, 9].

Research on TCM modernization has focused on explaining its mechanisms of action using molecular biological techniques on several targets; nevertheless, there have not been significant breakthroughs.

With the development of microbiology, intestinal microecology has become an intense area of research. The intestinal flora may play an active role in the host defense system by regulating mucosal local and systemic immunity and affect the lungs through interaction with the host [10, 11]. In the present study, *Yinlai* decoction was used to treat LPS-induced pneumonia in young rats to identify the effect of *Yinlai* decoction on pneumonia-related inflammation and intestinal flora.

## 2. Methods

### 2.1. Reagents and Instruments

Ordinary feed was purchased from Beijing Vital River Laboratory Animal Technology Co., Ltd. (Beijing, China). LPS was derived from *Escherichia coli* 055: B5 (batch number: 046M4045 V, Sigma, St. Louis, MO, USA). The rat single factor test kits, including interleukin (IL)-6 (A311125), tumor necrosis factor (TNF)-*α* (A311129), and IL-12 p40 (B311169), were from QuantoBio (Beijing, China). The Quant-iTPicoGreends DNA assay kit was from Thermo Scientific (P11496, Waltham, MA, USA). The TruSeq Nano DNA LT Library Prep Kit was from Illumina (San Diego, CA, USA). Instruments such as the NE-C900 compression atomizer (OMRON, Kyoto, Japan), refrigerated centrifuge (Eppendorf, Hamburg, Germany), Agilent 1290 UPLC (Agilent, Santa Clara, CA, USA), Agilent 6540 Q-TOF (Agilent, Santa Clara, CA, USA), and microplate reader (FLx800, BioTek, Winooski, VT, USA) were used.

### 2.2. Preparation of Yinlai Decoction

The crude herbs of the *Yinlai* decoction including *FlosLonicerae Japonicae* 30 g, *Semen Raphani* 10 g, *Fructus Forsythiae* 15 g, *Herba Houttuyniae* 20 g, *Radix Peucedani* 10 g, *Radix Scutellariae* 10 g, and *Fructus Trichosanthis* 10 g were purchased from the Guoyitang Outpatient Clinic of the Beijing University of Chinese Medicine (Beijing, China). These herbs were combined, soaked in 1 L of pure water for 30 min, and boiled for 75 min in the 4^th^ position of the decocting machine. This material was filtered, and the dregs were removed. The remaining material was cooled to room temperature, and pure water was added to the final volume of 393 mL, mixed, and stored at 4°C.

Component analysis of *Yinlai* decoction was performed using an Agilent 1290 UPLC and an Agilent 6540 Q-TOF. The liquid chromatogram conditions were an Agilent Proshell 120 EC-C18 column (3.0 × 150 mm, 2.7 *μ*m) and the mobile phase was a gradient of 0.1% formic acid-water and formic acid-methanol at the elution sequence of 0–2 min (15%–45% B), 2–8 min (45%–72% B), 8–25 min (72%–95% B), and 25–30 min (95%–100% B), at a column temperature of 35°C and a flow rate of 0.6 mL/min. The component analysis of *Yinlai* decoction is shown in Supplementary Figure 1 and Supplementary Table 1.

### 2.3. Animals and Sample Preparation

Thirty specific pathogen-free Sprague–Dawley male rats (four weeks old, weighing 110 ± 10 g) were purchased from Beijing Vital River Laboratory Animal Technology Co., Ltd. They were housed in the animal room of the Beijing University of Chinese Medicine in nonbarrier environments with natural light. After adaptive feeding for three days, they were randomly divided into three groups, each containing ten in each group: the N group, the P group, and the PT group. Mice were kept in group cages (ten mice per cage). In the P and the PT groups, the rats inhaled LPS (0.5 mg/mL, 5 mL, 30 min/day), and the N group was treated with atomized pure water (5 ml, 30 min/day). The success criterion of the pneumonia model was based on the lung pathology using hematoxylin-eosin (H & E) staining. In the PT group, *Yinlai* decoction (2 mL/100 g) was administered intragastrically daily, while in the N and P groups, pure water (2 mL/100 g) was administered intragastrically.

After three days, the rats were fasted for 12 h and then anesthetized using 2% pentobarbital sodium (0.1 mL/10 g). The maximum percentage of body weight loss was 17.3% because of fasting. Blood was collected from the abdominal aorta, centrifuged at 3000*g* for 10 min, and the serum was stored in a –80°C refrigerator. Following the principle of euthanasia, animals were decapitated after blood collection. The lung tissues and feces in the colon were also collected. The lung tissues were soaked in 10% formalin and stored at 4°C. The feces were frozen quickly in liquid nitrogen and stored at –80°C.

### 2.4. Visceral Index and Histological Analysis

Daily changes in body weight were recorded. After obtaining the samples, the spleen and thymus were collected, washed in normal saline, and weighed after drying. The visceral index was calculated as visceral weight/animal mass × 100%. The lung tissues in formalin were embedded in paraffin and sectioned. The sections were stained with H & E for histological observation under a light microscope.

### 2.5. Measurement of Serum Inflammatory Factors Using AimPlex Kits

The serum was centrifuged at 10000*g* for 4 to 5 min at 4°C, and the supernatants were removed. The standards and samples were prepared according to the manufacturer's instructions. In the experimental wells, 45 *μ*l/well of hybrid microspheres were added with 45 *μ*L/well of sample or standard, and shaken away from light for 60 min; 100 *μ*L/well of 1 × washing buffer was added and washed three times. 25 *μ*L/well of 1 × Biotin-dAb was added and shaken away from light for 30 min. These procedures were repeated and washed three times; 25 *μ*L/well of 1 × SA-PE was added and shaken away from light for 20 min; 100 *μ*L/well of 1 × washing buffer was added and washed twice. 60 *μ*L of remaining microspheres were mixed with 200–300 *μ*L of 1 × reading buffer for testing. A flow cytometer was used for detection, and the data were processed using FCAP Array 3.0 software.

### 2.6. 16S rDNA Measurement in Intestinal Flora

We entrusted Beijing Weishengtai Technology Co., Ltd. to finish the testing. The total DNA of the fecal samples was extracted, and the DNA extraction quality was measured using 0.8% agarose gel electrophoresis. The DNA was quantified using an ultraviolet spectrophotometer. The primers were designed according to the conserved regions in the sequence, and the sample-specific barcode sequence was added. Then, the variable regions of the rRNA gene (single or multiple in consecutive) or specific gene fragments were subjected to polymerase chain reaction (PCR) amplification. The amplified product was detected using 2% agarose gel electrophoresis, and the target fragments were subjected to gel extraction. Based on the preliminary quantitative results of electrophoresis, the PCR amplified product was performed using fluorescence quantification. Then, each sample was mixed in a ratio according to the sequencing requirement. High-throughput sequencing was performed, and the optimal sequencing length of the target fragment was 200–450 bp.

### 2.7. Statistical Analysis

All data results were rounded to two decimal places. The analyst was blinded to the conditions of the test groups. The statistical analysis was conducted using SPSS 25.0 (IBM, New York, NY, USA). If the data were normally distributed, a one-way analysis of variance was performed for intergroup comparisons. For the homogeneity of the variance, the least significant difference test was used; if not, the Thamnini T2 (*M*) test was performed. If the data were not normally distributed, the nonparametric Kruskal–Wallis was used for comparisons among groups. The data were expressed as the mean ± standard deviation. *P* < 0.05 was considered statistically significant.

## 3. Results

### 3.1. Yinlai Decoction Attenuates LPS-Induced Pulmonary Inflammation and Serum Inflammatory Factor Levels

**Figure 1:**
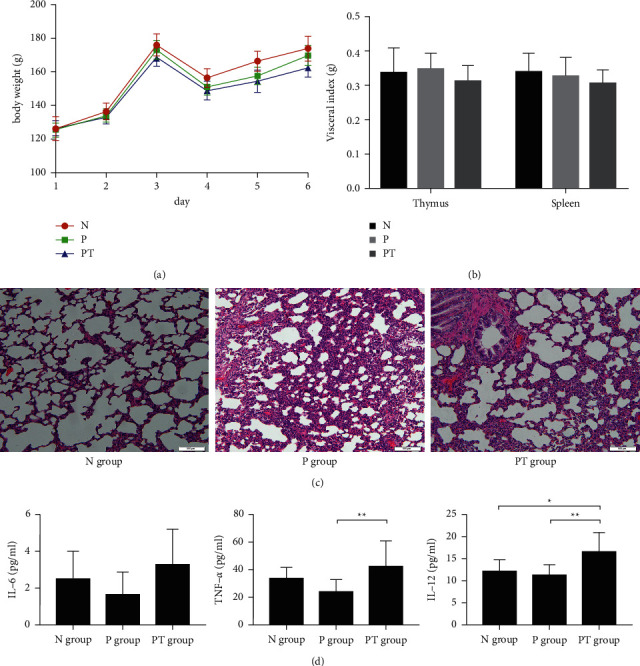
*Yinlai* decoction attenuates LPS-induced pulmonary inflammation and serum inflammatory factor levels. (a) Changes in body weight. (b) Thymus and spleen index. (c) H & E staining in lung tissue (200x. (d) Serum inflammatory factor levels. The data are expressed as the mean ± standard deviation (*n* = 8 per group). ^*∗*^*P* < 0.05 and ^*∗∗*^*P* < 0.01 with comparisons indicated by lines. Yinlai decoction regulates the overall intestinal flora structure of rats with LPS-induced pneumonia.

The component analysis of the *Yinlai* decoction is shown in Supplementary Figure 1 and Supplementary Table 1. 22 compounds were identified from Yinlai Decoction. There were no adverse events in either experimental group. The body weights of the three groups showed increasing trends. There was no significant difference in body weight among the three groups on the first three days. On the fourth day, LPS was added to pneumonia induction in the pneumonia group (P) and *Yinlai* decoction group (PT). A *Yinlai* decoction was provided to the PT group. The body weights of rats were lower in the P and the PT groups than in the blank control group (N) (Figure 1(a)). There were no significant differences in thymus and spleen indexes among the three groups (Figure 1(b)). H & E staining showed that, compared with the N group, the lung tissue structure was damaged, the alveolar septa showed different sizes, the alveolar walls were thickened or fractured, and there was a large amount of neutrophil infiltration and erythrocyte extravasation in the interstitial lung and proliferation of the vascular endothelial cells in the P group. Compared with the P group, the lung tissue and alveolar structure in the PT group were clearer, and there was less pulmonary interstitial hyperplasia and only mild inflammatory infiltration (Figure 1(c)). The levels of IL-6, IL-12, and TNF-*α* in the P group were not significantly different from the N group; however, there was a descending trend. Compared with the P group, IL-6 levels in the PT group showed a rising trend, and IL-12 and TNF-*α* levels were significantly higher in the PT group. IL-12 levels in the PT group were significantly different from those of the N group (Figure 1(d)).

**Figure 2:**
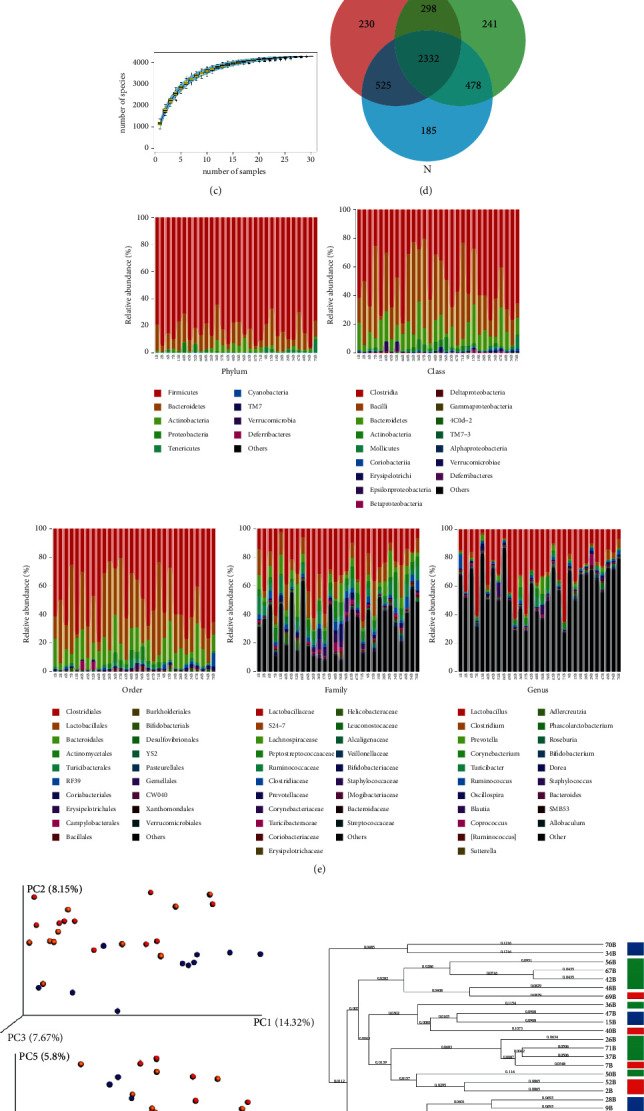
Structural changes of the overall intestinal flora in LPS-induced pneumonia rats with after *Yinlai* decoction. (a) Distribution of sample sequence length. (b) Shannon diversity curve. (c) Species accumulation curves. (d) Vennn diagrams of OTUs shared among groups. (e) Microbiological group statistics at each taxonomic level following phylum, class, order, family, and genus. (f) PCA and PCoA analysis of samples. The closer the distance between the two points, the higher the similarity of microbial community structure between the two samples. (g) Clustering tree analysis indicates the shorter the branch length becomes, the more similar the two samples are.

We analyzed the structural changes of the intestinal flora of the three groups. A total of 1,575,096 available reads and 4,289 operational taxonomic units (OTUs) were obtained from 30 samples. The sequencing length was distributed between 400 and 450 bp (Figure 2(a)). Species accumulation curves and Shannon diversity curves tended to be steady, indicating that the sequencing results were appropriate for reflecting the diversity of the current sample (Figures 2(b) and 2(c)). The Venn diagram showed that 2,332 OTUs were shared among the three groups; 2,857 in the N and P groups, 2,630 in the P and PT groups, and 2,810 in the N and PT groups (Figure 2(d)).

Histograms of gut microbial community structures revealed the distribution of microbial populations at each taxonomic level (Figure 2(e)). At the phylum level, samples of the three groups contained *Firmicutes*, *Bacteroidetes*, *Actinobacteria*, *Proteobacteria*, Tenericumes, *Cyanobacteria*, TM7, Verrucomicrobia, and *Deferribacteres*, of which the abundances of the *Firmicutes* and *Bacteroidetes* were the highest, although without statistical significance among the three groups. Ultimately, 85 genera were identified in the three groups, of which unclassified *Clostridiales* and *Lactobacillus* accounted for the majority, but without significant differences among the three groups. The abundances of *Corynebacterium*, *Jeotgalicoccus*, *Staphylococcus*, and *Aerococcus* in the P group were significantly higher than in the N and PT groups. Principal component analysis and principal coordinates analysis showed that the intestinal flora of the P group was altered compared with the N group (Figure 2(f)), and the intestinal flora of the PT group did not return to the normal level (N group); however, the system clustering tree demonstrated that the level of the PT group was closer to that of the N group (Figure 2(g)).

### 3.2. Yinlai Decoction Regulates the Growth of Specific Intestinal Flora

**Figure 3:**
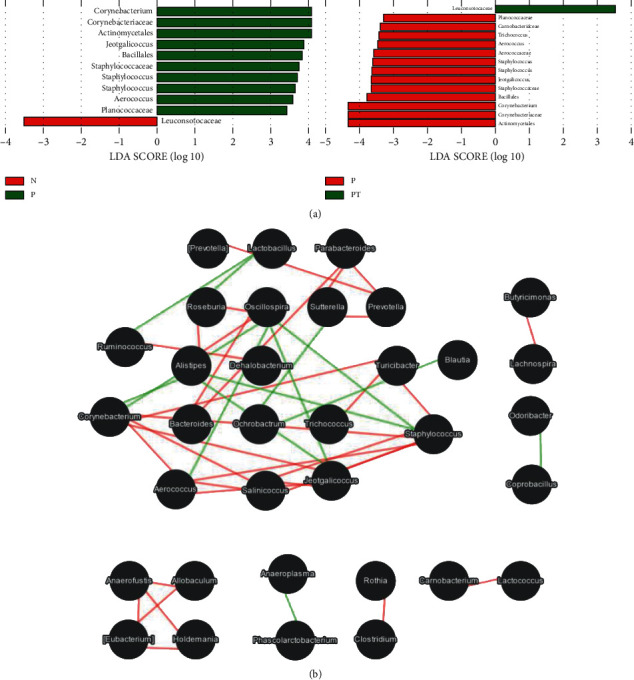
Comparison of differential microflorae between LPS-induced pneumonia rats after the intervention of *Yinlai* decoction. (a) A bar chart of microbial communities with significant differences among groups. The length of the bar graph is positively related to the significance of the difference. (b) Spearman rank correlation of the top 50 genera with statistical significance. The nodes represent each significant genus. A red line indicates a positive correlation and a green line indicates a negative correlation.

The differences in the intestinal microflora were analyzed using LEfSe among the three groups. In comparing the N and P groups, ten florae were significantly correlated in the P group, including *Corynebacterium*, *Corynebacteriaceae*, Actinomycetales, *Jeotgalicoccus*, *Bacillales*, *Staphylococcaceae*, *Staphylococcus* in Staphylococcaceae, *Staphylococcus* in *Planococcaceae*, *Aerococcus*, and *Planococcaceae*. *Leuconostocaceae* was significantly correlated in the N group. For comparisons between the P and PT groups, similar microflora characteristics were displayed with the N group. *Trichococcus* and *Carnobacteriaceae* were identified as new significantly related florae in the P group. There was no statistical significance in microbial population distribution between the N and PT groups at each taxonomic level (Figure 3(a)).

Flora function was analyzed using Spearman correlation networks of the dominant microbial interactions among groups; these indicated synergistic relationships among *Corynebacterium*, *Jeotgalicoccus*, *Staphylococcus*, and *Aerococcus*, all or some of which competed with *Oscillospira* and *Alistipes* but did not have a direct competitive relation with Lactobacillus (Figure 3(b)).

## 4. Discussion

The abuse of antibiotics leads to bacterial resistance, increases the difficulty of diagnosis and treatment of pneumonia, and carries substantial side effects such as flora disorder [12, 13]. TCM has aroused substantial attention as an alternative. Studies of TCM modernization have generally confirmed that Chinese herbal medicines possess antiviral effects against HINI, RSV, and others [14–16]. However, there have been few studies of respiratory inflammation caused by bacterial infection. Long before Western medicine was introduced in China, Chinese herbal medicines had been used to treat respiratory infections, but without specific differentiation on the types of pathogens contracted. The role Chinese herbal medicine plays in the treatment of bacterial pneumonia is unclear. In addition to directly killing pathogens, reducing inflammation, and improving immunity, it remains unclear what other properties Chinese herbal medicine possesses.

Bacteria are the most common pathogens causing pneumonia. LPS is present on the surface of Gram-negative bacteria and acts as an antigen to induce inflammation [17]. In the present study, we established a rat model of LPS-induced pneumonia. LPS combined with specific molecules activated the intracellular inflammatory signaling pathway, which was stable and simulated the host inflammatory immune response during pneumonia. We found that LPS-induced pneumonia model rats presented impaired lung tissue structures, different sizes of alveolar septa, thickening or fracture of the alveolar walls, abundant neutrophil infiltration, erythrocyte extravasation in the pulmonary interstitium, and vascular endothelial cell proliferation. Compared with the N group, serum IL-6, IL-12, and TNF-*α* in the rats displayed a descending trend.

Studies have found that the main compounds identified from *Yinlai* decoction, such as caffeic acid, raphanin, and baicalin, have anti-inflammatory and immune regulation effects [18–20]. We found that with treatment with *Yinlai* decoction, lung tissue and alveolar structure became clear, interstitial hyperplasia was reduced, and there was only mild inflammatory infiltration. Levels of IL-12 and TNF-*α* were significantly elevated. These findings suggest that LPS successfully simulated the pathological state of pneumonia, and *Yinlai* decoction alleviated the pulmonary inflammation and improved systemic inflammation.

There is a growing body of evidence to suggest a correlation between respiratory diseases and intestinal flora, especially infectious diseases [21, 22]. Pulmonary infections also result in changes in the gut microbiota. Intestinal microorganisms in mice are composed of *Firmicutes* and *Bacteroides*, and the balance between the two can be disturbed in several diseases. Groves et al. found that the relative abundance of *Bacteroides* in the intestinal flora of mice after RSV infection significantly increased, and that of *Firmicutes* decreased accordingly [23]. Studies have found that LPS directly applied to the lungs of healthy mice caused acute lung injury and that bacterial loads of the lungs and blood were synchronized, suggesting that the bacteria temporarily translocated into the circulation and caused acute increases of bacterial loads in the cecum. Antibiotic treatment can eliminate bacterial translocations but does not affect the cecum [24].

This study showed that the intestinal flora of rats with LPS aspiration pneumonia was disordered, and the structure of the flora deviated from that of the N group. There were two and 15 significant differences at the levels of the phylum and genus, respectively. The abundances of *Corynebacterium*, *Actinomycetales*, *Jeotgalicoccus*, *Bacillales*, *Staphylococcus*, *Aerococcus*, *Planococcaceae*, and *Trichococcus* were elevated.

In recent years, *Corynebacterium* has been repeatedly reported to cause pulmonary infections, and the detection rate of *Corynebacterium striatumis* was the highest [25–27]. Researchers have come to realize that *Corynebacterium* can infect the host as a conditioned pathogen and can be symbiotic with common pathogens [28]. Because it is a multidrug-resistant strain, *Corynebacterium* is not sensitive to antibiotics such as penicillin, cefotaxime, or ciprofloxacin, which predisposes the host to secondary infections after conventional antibiotic treatment [29].

*Staphylococcus* is an important pathogen in humans. It causes extensive infections after inhalation or infected blood when human immunity declines. In patients with bacterial pneumonia caused by *Staphylococcus aureus*, the mortality rate reached 24%–50% [30, 31].

*Aerococcus* is a conditioned pathogen and is highly susceptible to infection in patients with poor immunity, open trauma, burns, and susceptibility to drug resistance. *Bacillus* is an aerobic or facultative anaerobic Gram-positive bacterium. *Bacillus anthracis* is a pathogen that infects humans and animals and is highly pathogenic by infecting the host through the skin, mucus, and respiratory inhalation. *Bacillus cereus* causes systemic diseases such as food-borne diseases, chronic skin infections, meningitis, and pneumonia [32, 33]. Central venous catheters were associated in 98.8% of patients with *Bacillus* bacteremia, and central venous catheterization was an independent risk factor for bacteremia [34].

The intestine is the largest immune organ in humans. There are hundreds of millions of microorganisms in the intestine, maintaining homeostasis with the host. Intestinal flora regulates pulmonary immune responses [35–37]. Alveolar macrophages are thought to be the first line of defense when pathogens invade the lungs. In the host defense system, intestinal microbes bolster the host's defense against pneumococcal pneumonia by enhancing alveolar macrophage function. Bacteria in C57BL/6 mice, after removing intestinal flora, spread rapidly, accompanied by severe inflammation and organ failure. Mortality increased after contracting streptococcal pneumonia accompanied by decreased phagocytosis of alveolar macrophages and an inadequate response to LPS; this finding suggested that the intestinal flora might protect the host against pneumococcal disease [38]. In influenza virus-infected mice, similar results were obtained, including impaired innate and adaptive antiviral immune responses in mice with dysbacteriosis, prolonged virus clearance, and reduced control of viral replication [39].

These findings suggest that the intestinal flora might serve as a target for pneumonia treatment. Researchers are attempting to find strong evidence for anti-infective regimens such as microecological preparations and fecal microbiota transplantation [40, 41]. Several clinical studies have confirmed that probiotics may reduce the risk of contracting ventilator-associated pneumonia in intensive care unit patients by reconstructing normal flora or inhibiting colonization with drug-resistant bacteria [42]. *Lactobacillus rhamnosus* GG and *Bifidobacterium longum* alleviated lung damage after sepsis [43]. Although probiotic use is known to treat pulmonary inflammation and even systemic infection by improving gastrointestinal barrier function, increasing antimicrobial peptides of host cells, regulating intestinal flora constitution, and reducing bacterial translocation, potential risks of using microecological preparations remain [44].

Chinese herbal compounds are characterized as systemic and multitarget. Investigators have long attempted to measure their effectiveness and explore the mechanisms from the perspective of molecular biology. However, due to the complexity of the compounds, they cannot be replaced simply by using a single component, which has introduced difficulties for TCM modernization. In recent years, the development of microbiology has generated new opportunities for TCM modernization. The concept of “the lung is related to the stomach and intestines” has been recorded in TCM theory. It is believed that the lungs, stomach, and large intestine are closely related. On the one hand, if the lung diseases cannot be resolved, they are transmitted to the stomach and intestines over long periods, causing functional changes of the latter; on the other hand, adjusting the stomach and large intestine function can also prevent pulmonary diseases and realize the goal of “prevention first.”

The composition of *Yinlai* decoction is based on treating the lungs, the stomach, and the large intestine simultaneously. When treating the lung, the recovery of the stomach and large intestine function is also considered; *Semen Raphani* and *Fructus Trichosanthis* facilitate the alleviation of lung inflammation. Our findings in the present study suggest that *Yinlai* decoction restores the intestinal flora structure closer to that in normal rats and that it gradually returns to equilibrium. *Glucoraphanin*, the component of *Yinlai* decoction, can be metabolized into *sulforaphane* through intestinal flora. Research shows that *sulforaphane* exerts anti-inflammatory effects against LPS-induced acute lung injury in mice through the Nrf2/ARE pathway [45]. Intestinal flora may be an essential target for *Yinlai* decoction in the treatment of pneumonia.

The role of *Bifidobacteria* and *Lactobacilli* in maintaining human health and regulating immunity has been widely recognized. They are the most used intestinal probiotics [46, 47]. As shown by our results, *Yinlai* decoction did not significantly increase the abundance of *Bifidobacterium* and *Lactobacillus* but did change the abundance of *Leuconostocacea*e in *Lactobacillales*, *Aerococcaceae*, *Aerococcus*, *Carnobacteriaceae*, and *Trichococcus*, of which the abundance of *Leuconostocaceae* significantly increased. *Leuconostocmesenteroides* is an essential strain of *Leuconostocaceae*. Studies have found that *Lactobacillus plantarum* (Lp) and *Leuconostocmesenteroides* (Lm) inhibited H1N1 and H7N9 viral replication in the lungs of mice, prolonging survival and increasing survival rates [43]. However, due to the limitations of detection, 16S RNA has not been able to detect the level of microbial species. There is no direct evidence that *Yinlai* decoction promotes the growth of beneficial bacteria such as *Leuconostocmesenteroides*. Spearman correlation network analysis of dominant species interactions revealed a direct cooperative relationship among *Corynebacterium*, *Jeotgalicoccus*, *Staphylococcus*, and *Aerococcus* at the genus level, and they did not show any direct competition with *Lactobacillus*. We speculate that *Yinlai* decoction may help the intestinal flora to restore equilibrium by inhibiting the colonization of pathogenic bacteria and adjusting the ratio between probiotics and pathogenic bacteria.

## 5. Conclusions

The diversity of intestinal flora in rats with LPS-induced pneumonia decreased, and the structure of the flora was disordered. This lung inflammation was inhibited by *Yinlai* decoction treatment, which significantly increased serum levels of TNF-*α* and IL-12 and diversity of intestinal flora and balanced the floral structure. Intestinal flora may be an essential target for *Yinlai* decoction in the treatment of pulmonary inflammation. By inhibiting the colonization of pathogenic bacteria and adjusting the ratio between probiotics and pathogenic bacteria, *Yinlai* decoction maintains a dynamic equilibrium of intestinal flora. Whether its effect on pneumonia is mediated by interference with the structure of intestinal flora awaits further confirmation.

## Data Availability

The datasets generated or analyzed during the current study are available from the corresponding author on reasonable request.

## Supplementary Materials

Supplementary Materials Supplementary Figure 1. TIC diagram of *Yinlai* Decoction. Supplementary Table 1. 22 identified compounds from *Yinlai* Decoction.
