# Supplementary material for: Intestinal Flora: A Potential Mechanism by Which Yinlai Decoction Treats Lipopolysaccharide-Induced Pneumonia
Source: Evid Based Complement Alternat Med. 2022 Mar 23;2022:3034714. doi: 10.1155/2022/3034714 (PMC8967558; doi:10.1155/2022/3034714)
Supplement: Supplementary Materials — Supplementary Figure 1. TIC diagram of Yinlai Decoction. Supplementary Table 1. 22 identified compounds from Yinlai Decoction. [file 3034714.f1.docx]

**Intestinal flora: a potential route of** ***Yinlai* Decoction to treat**

**lipopolysaccharide-induced pneumonia**

JINGNAN XU2+, XUEYAN MA1+, CHEN BAI1, XIN JIANG2, LING HUANG1, FEI GAO1, YINI LI1, HE YU1, TIEGANG LIU1*, XIAOHONG GU1*


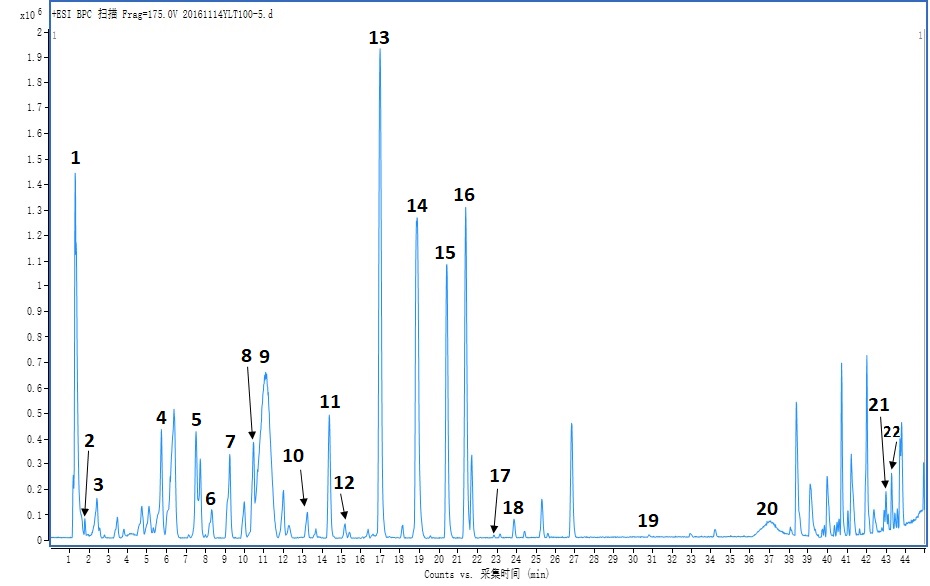


Supplementary Figure 1. TIC diagram of *Yinlai* Decoction.

Supplementary Table1. 22 identified compounds from *Yinlai* Decoction.

| No. | tR (min) | Molecular  formula | Molecular Mass(m/z) | Product ions  (m/z) | Diff  (ppm) | Compound  name |
| --- | --- | --- | --- | --- | --- | --- |
| 1 | 1.17 | C_9_H_8_O_4_ | 180.0523 | 203.0531[M+Na]^+^ | 3.45 | Caffeic acid |
| 2* | 1.81 | C_6_H_11_NOS_2_ | 178.0372 | 180.0511[M+2H]^+^ | 9.7 | Raphanin |
| 3 | 2.56 | C_17_H_14_O_6_ | 314.0828 | 315.0863[M+H]^+^ | 13.7 | Tricin |
| 4* | 5.73 | C_16_H_18_O_9_ | 354.0954 | 355.1055[M+H]^+^ | 8.7 | Chlorogenic acid |
| 5* | 7.46 | C_17_H_24_N_2_O_5_S | 368.1406 | 310.1530[M -CNS+H]^+^ | -18.2 | Sinapine thiocyanate |
| 6 | 8.36 | C_16_H_22_O_9_ | 358.1264 | 359.1292[M+H]^+^ | -12.5 | Sweroside |
| 7 | 9.21 | C_12_H_21_NO_10_S_3_ | 435.2044 | 437.2189[M+2H]^+^ | 16.3 | Glucoraphenin |
| 8 | 10.34 | C_24_H_26_O_7_ | 426.1039 | 427.1044[M+H]^+^ | 17.1 | Praeruptorin B |
| 9 | 10.67 | C_28_H_48_O_6_ | 480.2401 | 481.2436[M+H]^+^ | -7.7 | Brassinolide |
| 10* | 13.61 | C_27_H_30_O_16_ | 610.1543 | 611.1672[M+H]^+^ | -10.7 | Rutin |
| 11* | 14.62 | C_21_H_20_O_11_ | 448.0896 | 449.0893[M+H]^+^ | 18.0 | Galuteolin |
| 12 | 15.33 | C_12_H_23_NO_10_S_3_ | 437.0983 | 438.1026[M+H]^+^ | 7.5 | Glucoraphanin |
| 13 | 16.93 | C_15_H_10_O_7_ | 302.0327 | 303.0377[M+H]^+^ | -9.2 | Quercetin |
| 14 | 18.85 | C_25_H_24_O_12_ | 516.1168 | 539.0966[M+Na]^+^ | -17.4 | 3,5-Dicaffeoylquinic acid |
| 15 | 20.42 | C_28_H_48_O_5_ | 464.3502 | 465.3611[M+H]^+^ | 7.8 | Castasterone |
| 16* | 21.40 | C_21_H_18_O_11_ | 446.0741 | 447.0765[M+H]^+^ | -12.0 | Baicalin |
| 17* | 22.89 | C_27_H_34_O_11_ | 534.1951 | 557.1793[M+Na]^+^ | -8.9 | Phillyrin |
| 18 | 24.35 | C_16_H_12_O_6_ | 300.0584 | 301.0601[M+H]^+^ | -16.9 | Chrysoeriol |
| 19 | 31.82 | C_15_H_10_O_5_ | 270.0427 | 271.0500[M+H]^+^ | -1.8 | Apigenin |
| 20 | 37.98 | C_21_H_22_O_7_ | 386.1766 | 387.1859[M+H]^+^ | 5.4 | Praeruptorin A |
| 21 | 42.92 | C_18_H_34_O_2_ | 282.1789 | 265.1703[M -H_2_O+H]^+^ | -19.1 | Oleic acid |
| 22* | 43.05 | C_29_H_36_O_15_ | 624.3609 | 625.3705[M+H]^+^ | 3.2 | Forsythiaside A |
